# Shaping the learning curve: epigenetic dynamics in neural plasticity

**DOI:** 10.3389/fnint.2014.00055

**Published:** 2014-07-07

**Authors:** Zohar Z. Bronfman, Simona Ginsburg, Eva Jablonka

**Affiliations:** ^1^The Cohn Institute for the History and Philosophy of Science and Ideas, Faculty of Humanities, Tel-Aviv UniversityTel-Aviv, Israel; ^2^School of Psychology, Faculty of Social Science, Tel-Aviv UniversityTel-Aviv, Israel; ^3^Natural Science Department, The Open University of IsraelRaanana, Israel

**Keywords:** epigenetics, DNA methylation, non-coding small RNAs, neural plasticity, learning curve

## Abstract

A key characteristic of learning and neural plasticity is state-dependent acquisition dynamics reflected by the non-linear learning curve that links increase in learning with practice. Here we propose that the manner by which epigenetic states of individual cells change during learning contributes to the shape of the neural and behavioral learning curve. We base our suggestion on recent studies showing that epigenetic mechanisms such as DNA methylation, histone acetylation, and RNA-mediated gene regulation are intimately involved in the establishment and maintenance of long-term neural plasticity, reflecting specific learning-histories and influencing future learning. Our model, which is the first to suggest a dynamic molecular account of the shape of the learning curve, leads to several testable predictions regarding the link between epigenetic dynamics at the promoter, gene-network, and neural-network levels. This perspective opens up new avenues for therapeutic interventions in neurological pathologies.

## Introduction

**Figure 1:**
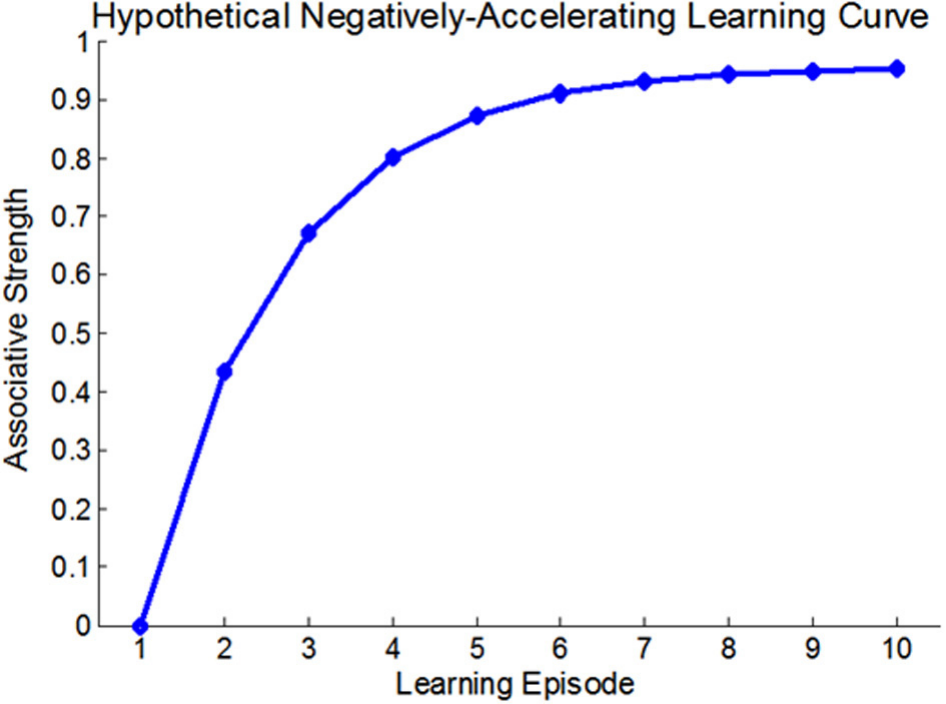
**A hypothetical negatively accelerating learning-curve; based on the Rescorla-Wagner associative learning model**. In this model, each learning-induced increment in associative strength is a fixed proportion of the difference between the current associative strength and the learning asymptote.

At the very outset of modern learning and memory research it was established that changes in behavior are usually acquired through a dynamic, gradual process of iterative experience, and that the relation between learning-strength and practice—i.e., the learning curve—is usually non-linear (Thorndike, 1898). A non-linear learning curve implies that the contribution of each learning-instance to the overall learning-strength is *state-dependent* rather than fixed (see Anderson, 2000 for many types of learning curves). For Example, the traditional Rescorla-Wagner associative learning model (Rescorla and Wagner, 1972), suggests that the associative strength increases as a fixed proportion of the difference between the current associative strength and its asymptote. Thus, this model predicts a negatively accelerating learning curve (see Figure 1). Although the vast majority of learning curves were described at the behavioral level it was realized that any behavioral manifestation of learning and memory entails some form of persistent neural plasticity. This suggested that there must be *neural learning curves* that reflect changes in neural plasticity as a function of practice. Several studies described such neural learning curves (Barnes, 1979; Cooke and Bear, 2010). For example, Cooke and Bear (2010) observed a diminishing neural learning curve in the visual cortex following a 5-day visual-exposure, showing that the stronger the current synaptic state, the smaller the increment upon future learning. These results are also consistent with most behavioral learning-curves (Anderson, 2000). However, although much has been learnt about the biochemical cascades underlying learning and memory, the molecular and cellular processes that render neural learning-dynamics state-dependent and determine the shape of the neural learning curves, are, as yet, poorly understood.

There are many different forms of long-term neural plasticity (persisting for more than 24 h). These include synaptic plasticity, which entails either changes in the efficacy of pre-existing synapses [for example, long-term potentiation (LTP; Bliss and Collingridge, 1993)] or the formation or elimination of synapses (Holtmaat and Svoboda, 2009); changes in somal depolarization (Kemenes et al., 2006) and even interactive and synergistic activity of both synaptic- and non-synaptic plasticity (Gao et al., 2012). In spite of the vast heterogeneity of mechanisms underlying long-term neural plasticity, *de-novo* transcription and translation are necessary processes common to all (reviewed in Leslie and Nedivi, 2011). The transcribed and/or translated gene products operate via diverse cellular mechanisms to modify neuronal properties that enable long-term neural plasticity such as the morphological changes taking place in the pre-synaptic axonal structures (Giuditta et al., 2002) and in the post-synaptic dendritic spines during long-term potentiation (LTP; a form of synaptic plasticity; Caroni et al., 2012). However, since there is constant turnover of proteins at synapses, it has been suggested that *epigenetic mechanisms*, which regulate experience-dependent gene expression and underlie its persistence are involved in maintaining functional neural states by supporting the persistent expression of the necessary RNAs and proteins (Miller and Sweatt, 2007; Zovkic et al., 2013).

## The involvement of epigenetic mechanisms in learning and memory

Persistent epigenetic changes are mediated through the operation of four types of interacting epigenetic mechanisms (Jablonka and Lamb, 2005/2014): (i) *Chromatin-marking*—whereby chromatin (the complex of DNA, proteins, and other components that constitute the chromosome) assumes different local and global conformations as it changes in response to signals. Examples of chromatin marks are methyl (CH3) groups added to cytosines in DNA (catalyzed by DNA methyltransferases, DNMTs) which, when present in regulatory regions, can repress transcription (Yu et al., 2001) and when oxidized into 5-hydroxymethylcytosine (5-hmC) correlates with transcriptional activation (Branco et al., 2012); histone post-translational modifications (PTMs) that render histones more or less accessible to transcription factors (Gräff and Tsai, 2013); histone variants (specific histone proteins that can displace the usual histones) that alter the conformation of chromatin and its accessibility to modifying enzymes; and non-histone proteins that are bound to DNA and are involved in chromatin marking, the regulation of chromatin condensation and topology, and the control or stabilization of other chromosomal functions (Talbert and Henikoff, 2010). (ii) *RNA-mediated gene silencing*—whereby silent states are initiated and actively maintained through repressive interactions between non-coding small RNA molecules and complementary regions in mRNA and DNA. Examples include microRNAs (miRNAs) and their complementary mRNA (Spadaro and Bredy, 2012) and piRNAs that are involved in the regulation of transcription in neurons, and long non-coding RNAs (Lasalle et al., 2013); in addition, small double-stranded RNAs can also activate genes (saRNAs), adding another layer of complexity to RNA-mediated regulation (Li et al., 2006). (iii) *Structural templating*—whereby pre-existing three dimensional cellular structures act as templates for the production of similar structures within the same cell or in daughter cells, such as the cellular inheritance of prions (Shorter and Lindquist, 2005). (iv) *Self-sustaining loops*—whereby a specific pattern of intracellular activity can be maintained when genes and their products form autocatalytic loops (Lisman et al., 2002).

During the last decade many studies showed that there are causal links between epigenetic mechanisms and long-term learning and memory. It was shown that learning induces both gene-specific and genome-wide (global) changes in neurons' epigenome (the cells' developmentally-established epigenetic state) and that manipulations of neural epigenetic activity, for example by catalyzing or inhibiting the activity of histone-modifying or DNA-methylating enzymes, affect learning and memory (for recent reviews see Blaze and Roth, 2013; Zovkic et al., 2013).

Gene-specific epigenetic changes were found in studies using rodent models of long-term neural plasticity. The majority of studies showed, as expected, that genes that support neural plasticity (i.e., genes whose expression levels increase following learning) are associated with positive regulatory epigenetic changes such as reduced DNA methylation and enhanced histone acetylation. Similarly, genes that inhibit the formation of memory are epigenetically suppressed following learning. For example, it was fond that several hours after training in the contextual fear conditioning paradigm (whereby a certain chamber is associated with an electric shock) rats' hippocampal neurons are demethylated at plasticity-facilitating genes such as the immediate early-gene *Zif268* (Miller et al., 2010), *reelin* (Miller and Sweatt, 2007) and *bdnf* (Lubin et al., 2008), while *PP1*, a learning-suppressing gene, becomes methylated (Miller and Sweatt, 2007). Thirty days after contextual fear conditioning, robust methylation of *calcineurin*, a suppressor of memory, was observed in cortical neurons (Miller et al., 2010), and several days after the extinction of this memory, hippocampal neurons are deacetylated (at H3) at the *c-Fos* promoter (Bahari-Javan et al., 2012). A similar pattern was observed in other forms of learning: 2 h after the extinction of cued-fear conditioning, there is an increase in histone H4 acetylation around the *BDNF* P4 gene promoter in the pre-frontal cortex (Bredy et al., 2007) and several hours after the induction of LTP, the *bdnf* and *reelin* genes in the medial pre-frontal cortex neurons are demethylated (Sui et al., 2012).

In addition to gene-specific changes, neuroepigenetic studies also show that there are genome-wide effects, which may represent an overall “aggregate” effect of learning. A survey of these studies (Bronfman et al., 2014) points to a positive correlation between increased global levels of histone acetylation, DNA methylation, and learning. Furthermore, manipulations of the enzymes that enhance global histone acetylation or global DNA methylation enhance the strength and persistence of learning, while a decrease in these enzyme activities decreases learning. These results appear to be consistent across taxa, time, brain-region, and learning task. Table 1 presents representative examples.

**Table 1:** **Genome-wide Epigenetic changes induced by learning**.

	**Genome-wide epigenetic changes**	**Time/Region/Task**	**Aminal**	**References**
**PTMs (HISTONE-TAIL ACETYLATION AND DI- AND TRI-METHYLATION)**				
1	Contextual fear conditioning increases acetylation of histone H3. Artificially elevating levels of histone acetylation *in vitro* enhances the induction of LTP	20 min–1	Rodents	Levenson et al., 2004
		Hippocampus		
		Contextual fear conditioning		
2	Increase in acetylation of Histones H3 and H4; HDAC inhibitors facilitate learning	3 h–Weeks	Rodents	Fischer et al., 2007
		Hippocampus and Cortex		
		Environmental enrichment; Associative learning; Spatial learning		
3	Increase in H3 acetylation across all regions of the hippocampus, while acetylation of lysine 9 on H3 is downregulated selectively in CA1. H4 acetylation is influenced in opposite directions in CA1 and DG, and is insensitive in CA3	1 h	Rodents	Castellano et al., 2012
		Hippocampus		
		Spatial memory		
4	Increase in H3 acetylation	15 min	Snails	Danilova et al., 2010
		Right parietal ganglion		
		Food aversion		
5	HAT (CBP/p300) activation (by CSP-TTK21) enhances memory (extending the time during which memory can be retrieved)	24 h	Rodents	Chatterjee et al., 2013
		Hippocampus		
		Spatial memory		
6	Overexpression of HDAC2 decreases dendritic spine density, synapse number, synaptic plasticity, and memory formation; HDAC2 deficiency results in increased synapse number and memory facilitation.	24 h	Rodents	Guan et al., 2009
		Hippocampus		
		Contextual fear conditioning; Spatial memory		
7	HDAC1 overexpression enhances extinction	Days	Rodents	Bahari-Javan et al., 2012
		Hippocampus		
		Contextual fear extinction		
8	Deletion of the *hda4*, a homolog of the mammalian *HDAC4* leads to enhanced memory, while the overexpression of this gene diminishes it	18–48 h	*C. elegans*	Wang et al., 2011
		The nervous system		
		Thermotaxic task		
9	HDAC inhibition enhances memory	2 h	Rodents	Bredy et al., 2007
		Pre-frontal cortex		
		Contextual fear extinction		
10	HDAC inhibition enhances memory	24 h	Rodents	Hawk et al., 2011
		Dorsal hippocampus		
		Object-location memory		
11	HDAC inhibition enhances memory	24 h	Rodents	Mahan et al., 2012
		Hippocampus		
		Contextual fear conditioning		
12	Increased H3 acetylation during reconsolidation; p300 HAT inhibitor impaired reconsolidation of strong memory; HDAC inhibitor enhances reconsolidation of a weak memory and an increase in histone H3 acetylation	1 h after consolidation	Crab	Federman et al., 2012
		Central brain		
		Context-signal memory reconsolidation		
13	Increase in trimethylation of H3K4 and dimethylation of H3K9	1–24	Rodents	Gupta et al., 2010
		Hippocampus		
		Contextual fear conditioning		
14	Increase in H3 di- and tri-methylation of histones	1–24 h	Rodents	Gupta-Agarwal et al., 2012
		Entorhinal cortex		
		Contextual fear conditioning		
15	H3S10 phosphorylation, H3K14 and H4K5 acetylation, and H3K36 trimethylation are increased rapidly (1 h–1 day) in the hippocampus, and remotely (1 day–7 days) in the PFC; Enhanced histone PTMs (by inhibition of *PP1*) facilitate memory, while inhibition of histone PTMs impairs memory	1 h–7 days	Rodents	Graff et al., 2012
		Hippocampus and PFC; Object memory		
16	Increase in H3 acetylation and DNMT3A expression; HDAC inhibitor enhances memory; DNMT inhibitor impairs memory	90 min	Rodents	Monsey et al., 2011
		Lateral amygdala		
		Cued fear conditioning		
**DNA METHYLATION**				
17	Total DNMTs, total HATs and global acetylation of H3 and H4 are elevated; infusion of DNMT inhibitor suppresses the induction of LTP (several hours) and interferes with trace fear memory (24 h); infusion of HDAC inhibitors enhances LTP (several hours) and trace fear memory (24 h)	2–24 h	Rodents	Sui et al., 2012
		Medial pre-frontal cortex		
		LTP; trace fear conditioning		
18	Enhanced DNMT expression after conditioning; blocking DNMT's activity abolishes memory	1 h	Rodents	Miller and Sweatt, 2007
		Hippocampus		
		Contextual fear conditioning		
19	Learning involves DNMT3 upregulation and, depending on treatment time, DNMT inhibition reduces the acquisition and retention of memory and alters its extinction	5 h	Honey bee	Lockett et al., 2010
		Mushroom bodies		
		Pavlovian olfactory discrimination and extinction		
20	Exposure of slices to DNMT inhibitor results in an immediate diminution of LTP	3 h	Rodents	Levenson et al., 2006
		Hippocampus		
		LTP (*in vitro*)		
21	Blocking DNMT activity impairs memory	2 h	Rodents	Lubin et al., 2008
		Hippocampus		
		Contextual fear conditioning		
22	Blocking DNMT activity impairs memory	1–30 days	Rodents	Miller et al., 2010
		Hippocampus; Dorsomedial pre-frontal cortex		
		Contextual fear conditioning		
23	Blocking DNMT activity impairs memory acquisition	24 h	Rodents	Han et al., 2010
		Hippocampus		
		Conditioned place preference		

The effects of the manipulations of enzymes that regulate DNA methylation described in the Table are in line with studies showing that knocking out Piwi genes, which contribute to DNA methylation, results in reduced long-term facilitation (LTF), whereas Piwi overexpression enhances it (Rajasethupathy et al., 2012). A study showing that a gain-of-function mutation in the *Mecp2* gene (a gene that produces a protein that binds to methylated DNA and contributes to the inhibition of transcription) increases its binding to methylated DNA and enhances learning (Li et al., 2011) also points in the same direction.

How can one explain these global, robust correlations in a highly context-sensitive functional system? The positive correlation between neural plasticity and global increase in histone acetylation seems natural, since this type of PTM renders the DNA, which is wound around the histones, more accessible to transcription factors, and hence more prone to activity-induced gene-expression supporting neural plasticity. However, DNA methylation is usually associated with gene silencing, especially in gene promoter regions (Yu et al., 2001)—a property that seems to be at odds with the overall positive relation of increased DNA methylation with enhanced learning and memory. Three explanations have been suggested to account for this correlation (i) that methylation leads to the repression of the synthesis of specific memory-repressors, and hence to improved learning (e.g., Yu et al., 2011; Sui et al., 2012); (ii) that increased methylation in the body of the gene is associated with increased transcriptional activity; and (iii) that DNMTs are involved in both DNA methylation and demethylation and its demethylating role explains why its level increases with learning (Chen et al., 2012). However, although such suggestions may explain some specific effects, they do not account very well for the correlation between *global* increase in DNA methylation and improved learning. It is not clear that the methylation of suppressor genes is more prevalent than demethylation of learning enhancing genes, that the demethylating role of DNMT I is more common than its methylating role, and that methylation in the body of the gene has a greater quantitative effect on overall methylation than the suppression of promoters by methylation.

We propose an elaboration and an extension of the first proposal, and suggest that the increased global levels of DNA methylation is related to the suppressor of learning-suppressing sequences, but that the class of suppressors are not protein coding genes but rather the genomically widespread clusters coding for precursor sequences of miRNAs (pre-miRNAs), which, when processed, interfere with the gene-expression necessary for plasticity. Our suggestion is consistent with a study showing that knock-out of *Dicer1* (a key enzyme in the biogenesis of miRNAs) in mice, led to improvement in a variety of learning tasks (Konopka et al., 2010). Further studies should investigate whether the correlation between global increase in DNA methylation levels and long-term learning and memory is indeed mediated by the suppression of pre-miRNAs, and if so by what means.

Another possible explanation for the positive correlation between DNA methylation levels and learning may be related to the central role of inhibition and pruning in learning. The increased levels of global DNA methylation may reflect lateral inhibition, whereby gene-activity in “competing” neural networks (i.e., neurons that are not involved in the specific learning) is being actively silenced so as to enable the efficient establishment of the relevant associations. Such a process may include the migration of positive regulators of DNMTs, such as piRNAs through exosomes from the focal network to the neighboring networks. Exosomes are vesicles measuring 50–90 nm contained in larger intracellular multivesicular bodies (MVBs), which are released from certain cells into the extracellular environment by MVBs fusing with the plasma membrane. Neurons are known to secrete exosomes (Lachenal et al., 2011; Lai and Breakefield, 2012). If this inactivation plays an important role in learning we predict that the global increase in methylation would be specific to formerly active (“competing”) neurons that *are not* directly participating in the learning process (i.e., not necessary for the formation of the specific memory), while the (relatively few) “relevant” neurons will exhibit more demethylation, especially in learning-related genes. This possibility is consistent with recent findings showing that global methylation levels in neurons increase throughout ontogeny (Lister et al., 2013) and may thus reflect the selection-processes during synaptic pruning (for early discussions see Changeux et al., 1973; Changeux and Danchin, 1976; Edelman, 1987).

Finally, and perhaps most intriguingly, the global effects involving methylation and acetylation may represent a mnemonic principle first suggested in the early 20th century by Semon (1921, 1923); discussed by Schacter (2001), where acquisition-encoding processes bear on changes in inter- and intra-network resonance dynamics, dynamics which are “reawakened,” expanded and modified upon retrieval. Under this conceptualization, global epigenetic effects, which are involved in the encoding and retrieval processes that occur during the episodes that follow the first exposure to the learning conditions, involve an increase in the number of encoding cells and hence in the expansion of the size and number of neural networks involved in learning. Hence, the changes occurring during learning can be best observed at the global level rather than only the gene or cell level.

Several predictions follow from these proposals: (i) negative correlation between increased DNA methylation and miRNAs abundance will be found in single neurons that exhibit plasticity; (ii) increased methylation will be found in neighboring neurons that interfere with focused learning; (iii) neighboring neurons will exhibit increased levels of piRNAs that positively regulate DNMT activity; (iv) the number of neurons that undergo epigenetic changes such as DNA methylation and histone acetylation will increase and spread during learning.

The studies summarized in this section strongly support the growing consensus that there is a complex, “bidirectional” relation between the epigenetic system and long-term neural plasticity. Learning induces multiple changes to neurons' epigenome, and at least some of these changes persist, thus enabling the necessary ongoing gene-expression that maintains the neural state. Upon future learning, each neuron's epigenome constrains or actively regulates the activity-induced gene-expression pattern and hence shapes future plasticity (Zovkic et al., 2013). This unique “time-bridging” involvement of the epigenetic system in neural plasticity implies that learning may be history-sensitive via epigenetic dynamics: the influence of future learning may be dependent, upon the cell-specific epigenetic state, which is in turn, a function of learning history.

The idea that present and past experience can alter the future susceptibility and responsiveness of synapses via diverse molecular mechanisms was suggested by Abraham and Bear (1996) who called this phenomenon metaplasticity, or “the plasticity of synaptic plasticity” (Abraham, 2008). For example, short exposures to environmental enrichment that do not enhance late-LTP (>24 h), facilitate it upon re-exposure 6 weeks later (Buschler and Manahan-Vaughan, 2012). Another example is weak (sub-threshold) induction of LTP that does not change synaptic efficacy, yet can alter future changes in plasticity, by mediating the processes of silent synapses unmasking (a process also referred to as “priming”; Ward et al., 2006). However, little is currently known about the factors that affect the shape and dynamics of neural learning-curves upon continuous (prolonged) learning acquisition, and many of the underlying mechanisms remain to be identified.

In spite of the appreciation of metaplasticity, the majority of studies linking epigenetic changes to enhanced learning focused on the relation between the “end-points” of a specific learning task and the corresponding end-point epigenetic states and did not address the epigenetic dynamics *during* learning acquisition. We therefore present simple models, describing how different patterns of epigenetic changes that occur throughout the learning process may determine the shape of the neural learning curve.

## Models of neural epigenetic learning-curves

Our proposal is based on the observation that epigenetic mechanisms possess “mnemonic” properties and can therefore lead to the dynamic, time-dependent, encoding, and storage of information during learning. The unique self-perpetuating biochemical activity of the interacting epigenetic mechanisms ensures that epigenetic patterns (e.g., patterns of histone modifications or of DNA methylation) persist long after the initial induction period (Fischer et al., 2007; Miller et al., 2010; Zovkic et al., 2013). Hence, unlike most of the genome, the epigenome is “history-sensitive,” in the sense that it changes as a function of learning and development over time. Moreover, the epigenome regulates future activity-induced gene-expression, thus causally determining the effect of experience on plasticity. This combination of properties suggests that epigenetic dynamics during learning may render learning state-sensitive.

We present a model based on the chromatin marking system of DNA methylation. Although other epigenetic systems may be as suitable, we chose DNA methylation because of the acknowledged persistence of DNA methylation marks (e.g., Cedar and Bergman, 2012; Smith and Meissner, 2013), because methylation marks can accumulate (e.g., Ibrahim et al., 2011; Lu et al., 2013), and because global increase in DNA methylation levels is consistently correlated with enhanced learning (Table 1). However, the model can easily be adapted to any other epigenetic mechanism, for example to the accumulation of acetylation of histones and to the more complex construction of activity-specific epigenetic modifications, involving, for example, various types of histone modifications. In the model we make the simplifying assumption that there is a linear relationship between levels of DNA methylation, gene expression, and synaptic plasticity. This assumption is made solely for illustrative purposes and relaxing it does not alter the model's predictions. For example, the model can describe learning that is dependent on crossing a certain epigenetic marking threshold (for example, a certain number of chromatin marks must be reached for transcriptional activation or inactivation). In this case, while the accumulation of chromatin marks may be gradual, the effect on learning will be abrupt and step-like.

**Figure 2:**
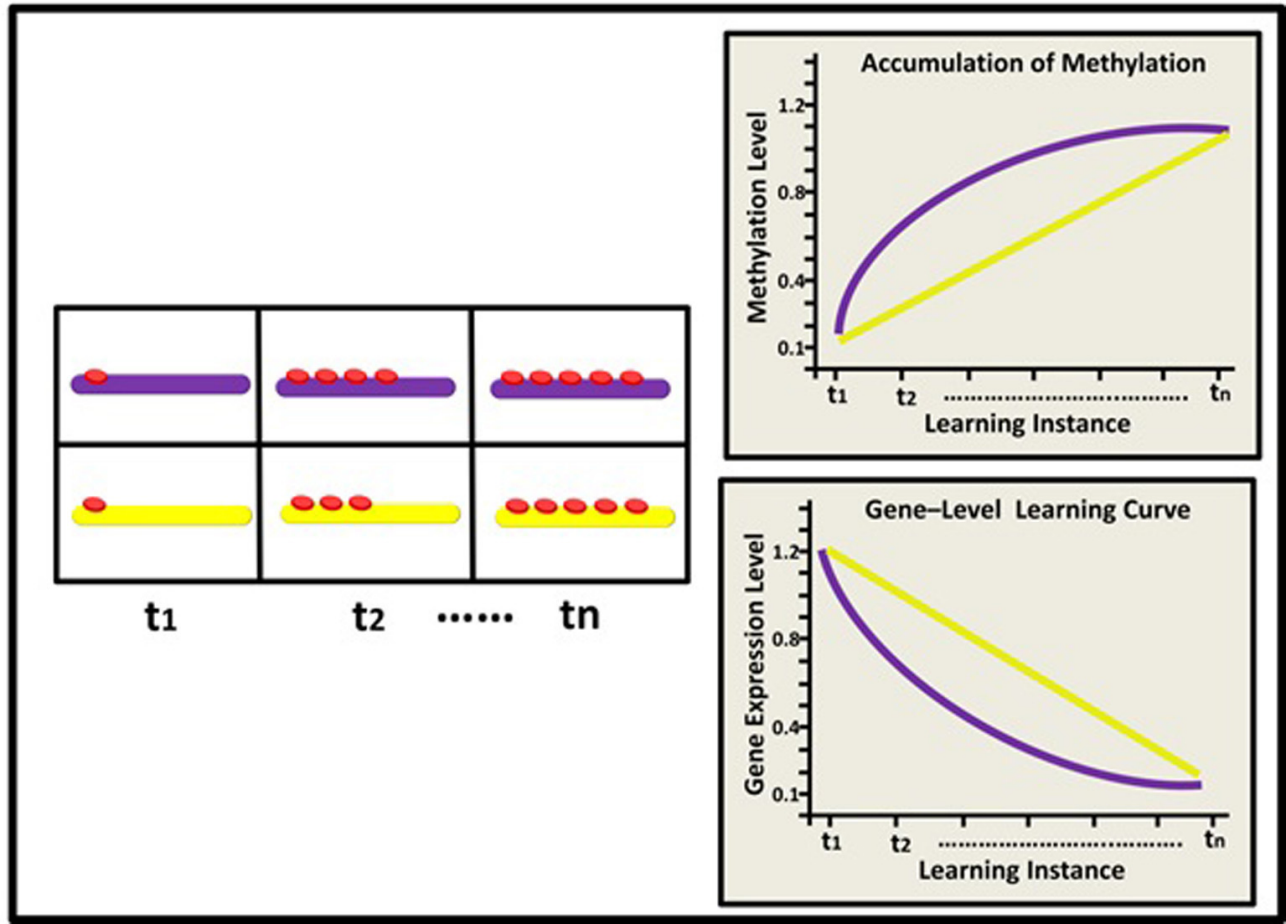
**Epigenetic dynamics during learning can shape the neural learning-curve at the single gene level: Initially (t1), two learning-suppressing gene promoters (purple and yellow rods) have similar basal methylation level (red buttons)**. During learning, (t2–tn) these two genes exhibit different patterns of epigenetic change and subsequently, different changes in expression levels. The purple gene exhibits a diminishing pattern of methylation marks accumulation (purple line; right upper panel) and therefore an inverse, diminishing “gene-expression learning curve” (purple line; right lower panel). The yellow gene exhibits a linear pattern of methylation accumulation and a linear learning curve (yellow line).

**Figure 3:**
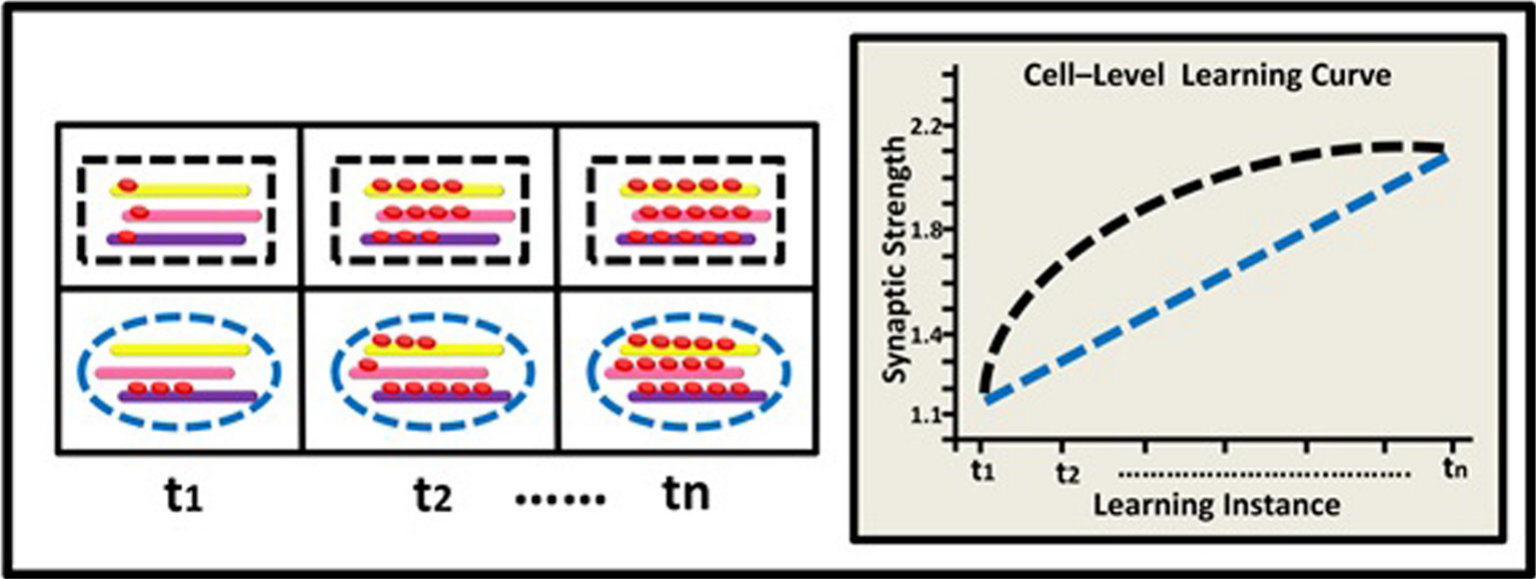
**Epigenetic dynamics during learning can shape the neural learning-curve at the single neuron's gene-network level**. At (t1), two types of neurons (black rectangle and blue ellipse) have similar overall basal methylation level (depicted by a 3-gene network: yellow, pink, and purple rods). During learning, (t2–tn) the two neurons exhibit different patterns of changes in their overall methylation level, their genes' expression profiles, and eventually their synaptic strength. The black neuron exhibits a diminishing pattern of overall methylation accumulation and a diminishing learning curve (black line) while the blue neuron exhibits a linear pattern of methylation accumulation and a linear learning curve (blue line).

**Figure 4:**
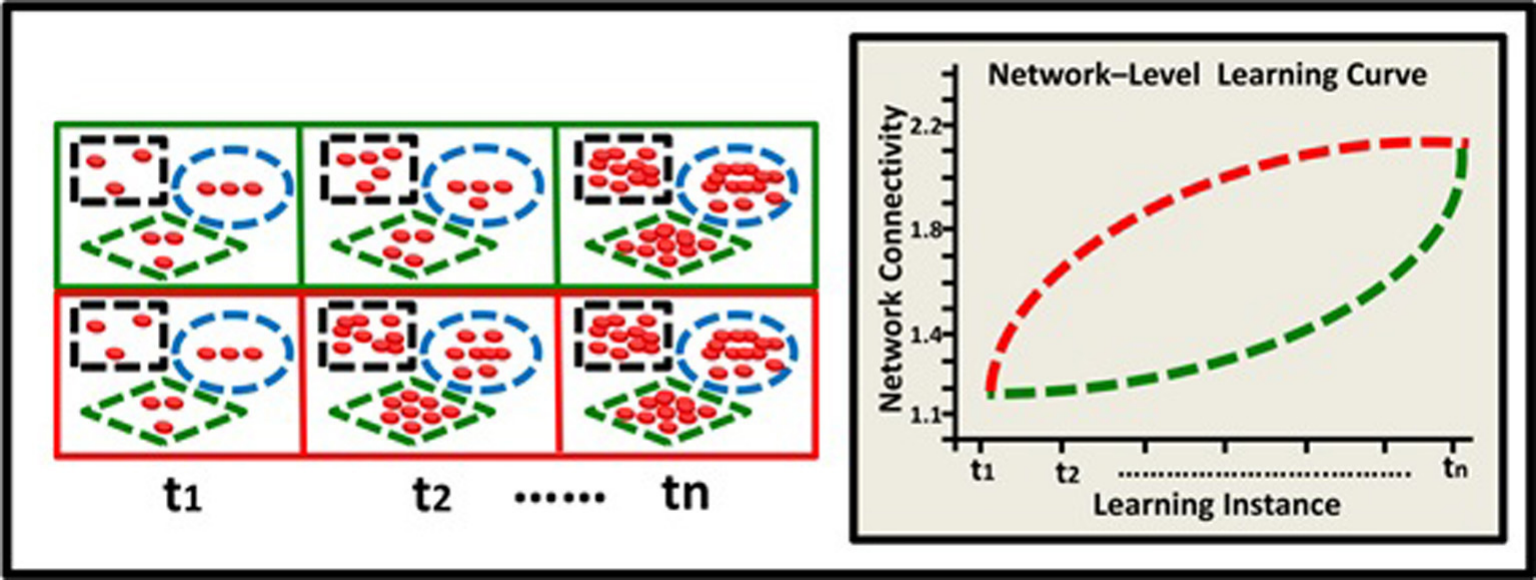
**Epigenetic dynamics during learning can shape the neural learning-curve at the neural-network level**. Two neural networks (green and red squares) comprised of 3 neurons each (black rectangle, blue ellipse, and green diamond), have similar overall basal methylation level at (t1). During learning, (t2–tn) these two networks exhibit different patterns of epigenetic change and subsequently different levels of overall methylation. The green network exhibits an accelerating pattern of overall methylation accumulation and therefore an accelerating learning curve (green line).The red network exhibits a diminishing pattern of methylation accumulation and therefore a diminishing learning curve (red line).

The model describes hypothetical kinetics of DNA methylation accumulation at three levels: the level of a suppressor single gene (Figure 2); the level of a gene network within a single neuron (Figure 3), and the level of a neural network involving three neurons (Figure 4). It shows how a specific pattern of accumulated epigenetic marks—a pattern we call the *epigenetic profile*—can eventually affect learning acquisition dynamics. At each level, two patterns are demonstrated, since we assume that epigenetic learning profiles are influenced by DNA sequence, cell type and ontogenetic stage, and hence may differ between genes, neurons, and brain regions (different brain regions indeed show different methylation patterns; Hata et al., 2013).

At the level of a single gene, we suggest that the manner by which methylation accumulates at the gene's promoter region during learning would determine the gene's expression level upon future learning (i.e., the gene's “learning curve”). Consider for example a *plasticity-suppressing* gene. In our model, each instance of learning is assumed to induce additional methylation marks at the gene's promoter region, down-regulating its expression—a process necessary for the manifestation and maintenance of plasticity. Importantly, different suppressor genes (or the same suppressor gene in different cells) may have different epigenetic profiles and thus exhibit different cumulative patterns. This would result in different expression levels during different states of learning acquisition, even if at the “end-point” of learning these genes may be similarly methylated (see Figure 2). Similar logic applies to learning-supporting genes, where progressive decrease in DNA methylation (demethylation) is expected during learning.

At the level of a single neuron, we focus on the *global* methylation level of the neuron's genome, illustrated here by the overall level of methylation in a gene-network comprised of 3 learning-related suppressor genes or gene regulators. We assume that as a result of learning, the neuron's global methylation increases. Note that the pattern of increase could differ between neurons: some neurons may exhibit non-liner increase in global methylation, whereas the pattern of changes in global methylation in other types of neurons could be linear (Figure 3).

Learning depends, of course, on complex networks involving interactions among many neurons. The observed alterations in epigenetic patterns as a result of learning may therefore reflect the *average change* at the neural network level, where different neurons may have different epigenetic learning profiles and hence contribute differentially to the observed aggregated effect. In a simplified 3-neuron-network (Figure 4) the learning curves show diminishing and accelerating changes in time in the averaged accumulation of DNA methylation within the entire network (across specific neurons and their underlying genes).

Behavioral learning curves and epigenetic effects are mostly described using average data. However, the shape of the learning curve may change when describing it at the individual (Gallistel et al., 2004) or at the trial-by-trial levels (Glautier, 2013) even to the extent of rendering it “threshold-like.” We suggest that the same logic applies to the presumed epigenetic kinetics: some epigenetic changes may seem as gradual, just as behavioral acquisition does, yet at finer granularity, epigenetic processes may also reflect (or affect learning in) a threshold-like process. The relations between gradual and discrete changes both in the epigenetic and the behavioral domains remain wide-open for future studies to investigate.

The model can be expanded to heed Semon's suggestion that networks expand during acquisition (Semon, 1921, 1923). Thus, rather than the number of chromatin marks on a single gene promoter increasing as a function of acquisition (Figure 2) it may be the case that the number of genes participating in learning increases with experience, and with it the number of the learning-linked chromatin marks we observe. Similarly, not only the number of marks on a single gene network (Figure 3), but the number of gene networks responsive to learning may increase with practice. Finally, and most importantly from this point of view, the accumulation of marks may occur not just within one pre-given network (Figure 4), but lead to the increase of the number of networks participating in learning and retrieval as a function of the number of learning episodes. The observation that the effects of learning in one area of the brain are “transferred” to additional areas (Graff et al., 2012) may support this conjecture. We believe that in addition to local cumulative epigenetic changes (such as genes, gene-networks, and neural networks; Figures 2–4) described in our model, learning also induces an expansion in the size and quantity of these factors.

The model can be tested by observing the epigenetic changes that occur *at several different points in time during learning acquisition*—and not just at the completion of the training protocol (as has been done in most studies), at the point in time where consolidation is thought to peak (e.g., Bousiges et al., 2010) or at several points in time *after* learning took place (e.g., Sui et al., 2012). Any standard neuroepigenetic learning paradigm, such as those reviewed in the first section and those mentioned above can be used to acquire these data^1^. We predict that such kinetic studies will show various patterns of *cumulative* epigenetic changes (such as decrease in methylation and increase in hydroxyl-methylation of learning-facilitating genes, increase in methylation of learning-suppressing genes and increase in global DNA methylation levels). Critically though, variations in these patterns of epigenetic dynamics should correspond to variations in behavioral learning curves. For example, we predict that following the induction of neural plasticity (via LTP, for example) one will find that: (i) different neurons (for example hippocampal vs. cerebellar) exhibit different neural learning-curves; (ii) these neurons manifest corresponding differences in their learning-induced epigenetic marks, such as patterns of changes in DNA methylation and histone acetylation; and (iii) manipulation of these neuronal epigenetic kinetics *during acquisition*, for example by using inhibitors of epigenetic enzymes, will affect the shape of their learning-curve.

How can epigenetic changes at the level of single genes, gene networks, or even neural networks predict change at the behavioral level? The relation between the behavioral and the molecular levels is clearly very complex, and we do not expect to see a simple mapping among the behavioral, neural, and epigenetic learning curves. However, as the review of the relations between epigenetic modifications and learning reviewed above has shown, in spite of the multiplicity of relations among different epigenetic factors and processes that underlie behavior, some robust correlations have been found between changes in the methylation of single genes and learning, and even more robustly, between more global changes and learning. We therefore believe that taking into consideration the kinetics of epigenetic changes as a function of learning may add to and enrich this picture.

If supported, we believe that our model may have implications for the interpretation and study of neurological diseases that show clear learning impairments, or of anxiety disorders, like post-traumatic stress disorder (PTSD), where memory regulation is specifically impaired (Zwissler et al., 2012; Zlomuzica et al., 2014). Accumulating evidence suggests that many neurological and neuropsychiatric disorders such as autistic spectrum disorder (ASD), Alzheimer's disease (AD) and schizophrenia (SZ) are associated with altered epigenetic activity. For example, ASD is associated with a decreased capacity for DNA methylation (Wu et al., 2010; Melnyk et al., 2012). Furthermore, some of the DNA methylation deficits in humans were recently found to be specific to brain-regions such as the temporal cortex and the cerebellum (Ladd-Acosta et al., 2013) that are associated with different forms of learning and memory. Similarly, AD is associated with epigenetic alterations in DNA methylation (Mastroeni et al., 2010) and abnormal micro-RNAs expression (Barak et al., 2013). Altered patterns of DNA methylation in immune system and nervous system development genes were found in PTSD (Uddin et al., 2010; Mehta et al., 2013), and schizophrenic patients show decreased genome-wide DNA methylation (Bonsch et al., 2012) and altered DNA demethylation mechanisms (Dong et al., 2012). Independently, these (and other) disorders were also characterized by abnormal learning curves: for example, ASD patients and animal models exhibit more moderate curves, (with slower acquisition rate), even when they ultimately reach, or are close to reach, control performance (Solomon et al., 2011; Walker et al., 2011). Although the methylation changes associated with AD, PTSD, and ASD have not been related to learning impairments, we believe that it may be worthwhile testing if some of these methylation changes influence their epigenetic learning curves. The epigenetics of PTSD in humans and of acute psychological stress in animal models of PTSD suggest that the epigenetic changes may contribute to the behavioral and cognitive effects of such psychological stresses (Yehuda and Bierer, 2009; Blaze and Roth, 2013). We speculate that in people suffering from different (global and trauma-specific) learning and memory impairments, learning acquisition dynamics may be found to be abnormal, with specific brain regions and epigenetic dynamics varying according to the syndrome. We also predict that task-specific learning curves that rely on different brain regions would be correlated with distinct regional epigenetic profile abnormalities, specifically in DNA methylation, hydroxymethylation and histone acetylation. Although learning impairments are only one facet of these syndromes, a better understanding of the dynamics of the epigenetics underlying them may uncover mechanisms and loci that escape detection with more static tests. If so, changes in the epigenetic learning curve may suggest possible novel avenues for therapeutic interventions by focusing on specific points *in time* for manipulating brain-region-specific epigenetic states.

## Conclusion

An overview of recent studies that investigate the involvement of the epigenetic system in long-term learning and memory reveals that the epigenome changes as a result of learning both at the level of specific genes and globally, across the entire genome (Blaze and Roth, 2013; Zovkic et al., 2013; Bronfman et al., 2014). Due to its mnemonic-like, self-sustaining activity (Miller and Sweatt, 2007; Ginsburg and Jablonka, 2009), the state of the epigenome can both represent neuronal history and affect future gene activity, thus bridging future and past learning. If this is indeed the case, we predict that epigenetic learning profiles that describe the manner by which the epigenome cumulatively changes as a function of learning will contribute to the shape of the cell's learning curve and to the overall learning curve of the neural networks that are involved in learning. We suggest that this profile could differ between tasks and brain regions and correspond to similar differences in behavioral learning curves observed for example in spatial learning (hippocampus) or eye-blink conditioning (cerebellum). Future studies should therefore investigate how differences in pattern of dynamic epigenetic change (especially DNA methylation, histone acetylation, and global microRNA profiles) correlate with differences in the shape of behavioral and neuronal learning curves, identify the factors that determine the neuronal epigenetic profiles and discover which manipulations result in changed learning curves.

Learning and the neural plasticity underlying it are cumulative and state-dependent processes. The epigenetic mechanisms that are involved in mediating, enabling and determining these dynamics are therefore of both theoretical and practical importance. The independent observations of abnormal learning curves and impairments in epigenetic processes in neurological pathologies such as ASD may reflect abnormal epigenetic profiles, and if so may open up new therapeutic possibilities. The future characterization of epigenetic dynamics during learning acquisition is therefore likely to be both fascinating and useful.

### Conflict of interest statement

The authors declare that the research was conducted in the absence of any commercial or financial relationships that could be construed as a potential conflict of interest.

## Footnotes

^1^A recent study showing that extinction of fear conditioning is associated with the accumulation of 5-hydroxycytosine (hmC, a chromatin mark associated with gene activity), provides the first example of such trial-dependent epigenetic dynamics (Li et al., 2014).

## References

[B1] Abraham W. C. (2008). Metaplasticity: tuning synapses and networks for plasticity. Nat. Rev. Neurosci. 9, 387 10.1038/nrn2356 18401345

[B2] Abraham W. C. Bear M. F. (1996). Metaplasticity: the plasticity of synaptic plasticity. Trends Neurosci. 19, 126–130 10.1016/S0166-2236(96)80018-X 8658594

[B3] Anderson J. R. (2000). Learning and Memory. New York, NY: John Wiley

[B4] Bahari-Javan S. Maddalena A. Kerimoglu C. Wittnam J. Held T. Bahr M. (2012). HDAC1 regulates fear extinction in mice. J. Neurosci. 32, 5062–5073 10.1523/JNEUROSCI.0079-12.2012 22496552 PMC6622110

[B5] Barak B. Shvarts-Serebro I. Modai S. Gilam A. Okun E. Michaelson D. M. (2013). Opposing actions of environmental enrichment and Alzheimer's disease on the expression of hippocampal microRNAs in mouse models. Transl. Psychiatry 3:e304 10.1038/tp.2013.77 24022509 PMC3784766

[B6] Barnes C. A. (1979). Memory deficits associated with senescence: a neurophysiological and behavioral study in the rat. J. Comp. Physiol. Psychol. 93, 74–104 10.1037/h0077579 221551

[B7] Blaze J. Roth T. L. (2013). Epigenetic mechanisms in learning and memory. Wiley Interdiscip. Rev. Cogn. Sci. 4, 105–115 10.1002/Wcs.1205 26304177

[B8] Bliss T. V. Collingridge G. L. (1993). A synaptic model of memory: long-term potentiation in the hippocampus. Nature 361, 31–39 10.1038/361031a0 8421494

[B9] Bonsch D. Wunschel M. Lenz B. Janssen G. Weisbrod M. Sauer H. (2012). Methylation matters? Decreased methylation status of genomic DNA in the blood of schizophrenic twins. Psychiatry Res. 198, 533–537 10.1016/j.psychres.2011.09.004 23102571

[B10] Bousiges O. Vasconcelos A. P. Neidl R. Cosquer B. Herbeaux K. Panteleeva I. (2010). Spatial memory consolidation is associated with induction of several lysine-acetyltransferase (histone acetyltransferase) expression levels and H2B/H4 acetylation-dependent transcriptional events in the rat hippocampus. Neuropsychopharmacology 35, 2521–2537 10.1038/npp.2010.117 20811339 PMC3055563

[B11] Branco M. R. Ficz G. Reik W. (2012). Uncovering the role of 5-hydroxymethylcytosine in the epigenome. Nat. Rev. Genet. 13, 7–13 10.1038/nrg3080 22083101

[B12] Bredy T. W. Wu H. Crego C. Zellhoefer J. Sun Y. E. Barad M. (2007). Histone modifications around individual BDNF gene promoters in prefrontal cortex are associated with extinction of conditioned fear. Learn. Mem. 14, 268–276 10.1101/lm.500907 17522015 PMC2216532

[B13] Bronfman Z. Ginsburg S. Jablonka E. (2014). The epigenetics of neural learning, in The Wiley-Blackwell Handbook on The Cognitive Neuroscience of Learning, eds Murphy R. Honey R. (New York, NY: Wiley-Blackwell).

[B14] Buschler A. Manahan-Vaughan D. (2012). Brief environmental enrichment elicits metaplasticity of hippocampal synaptic potentiation *in vivo*. Front. Behav. Neurosci. 6:85 10.3389/fnbeh.2012.00085 23248592 PMC3522088

[B15] Caroni P. Donato F. Muller D. (2012). Structural plasticity upon learning: regulation and functions. Nat. Rev. Neurosci. 13, 478–490 10.1038/nrn3258 22714019

[B16] Castellano J. F. Fletcher B. R. Kelley-Bell B. Kim D. H. Gallagher M. Rapp P. R. (2012). Age-related memory impairment is associated with disrupted multivariate epigenetic coordination in the hippocampus. PLoS ONE 7:e33249 10.1371/journal.pone.0033249 22438904 PMC3305324

[B17] Cedar H. Bergman Y. (2012). Programming of DNA methylation patterns. Annu. Rev. Biochem. 81, 97–117 10.1146/annurev-biochem-052610-091920 22404632

[B18] Changeux J. P. Courrege P. Danchin A. (1973). A theory of the epigenesis of neuronal networks by selective stabilization of synapses. Proc. Natl. Acad. Sci. U.S.A. 70, 2974–2978 10.1073/pnas.70.10.2974 4517949 PMC427150

[B19] Changeux J. P. Danchin A. (1976). Selective stabilisation of developing synapses as a mechanism for the specification of neuronal networks. Nature 264, 705–712 10.1038/264705a0 189195

[B20] Chatterjee S. Mizar P. Cassel R. Neidl R. Selvi B. R. Mohankrishna D. V. (2013). A novel activator of CBP/p300 acetyltransferases promotes neurogenesis and extends memory duration in adult mice. J. Neurosci. 33, 10698–10712 10.1523/JNEUROSCI.5772-12.2013 23804093 PMC6618502

[B21] Chen C. C. Wang K. Y. Shen C. K. (2012). The mammalian *de novo* DNA methyltransferases DNMT3A and DNMT3B are also DNA 5-hydroxymethylcytosine dehydroxymethylases. J. Biol. Chem. 287, 33116–33121 10.1074/jbc.C112.406975 22898819 PMC3460417

[B22] Cooke S. F. Bear M. F. (2010). Visual experience induces long-term potentiation in the primary visual cortex. J. Neurosci. 30, 16304–16313 10.1523/Jneurosci.4333-10.2010 21123576 PMC3078625

[B23] Danilova A. B. Kharchenko O. A. Shevchenko K. G. Grinkevich L. N. (2010). Histone H3 acetylation is asymmetrically induced upon learning in identified neurons of the food aversion network in the mollusk helix lucorum. Front. Behav. Neurosci. 4:180 10.3389/fnbeh.2010.00180 21151377 PMC2996247

[B24] Dong E. Gavin D. P. Chen Y. Davis J. (2012). Upregulation of TET1 and downregulation of APOBEC3A and APOBEC3C in the parietal cortex of psychotic patients. Transl. Psychiatry 2:e159 10.1038/tp.2012.86 22948384 PMC3565208

[B25] Edelman G. M. (1987). Neural Darwinism: The Theory of Neuronal Group Selection. New York, NY: Basic Books 10.1126/science.240.4860.1802 17842436

[B26] Federman N. Fustinana M. Romano A. (2012). Reconsolidation involves histone acetylation depending on the strength of the memory. Neuroscience 219, 145–156 10.1016/j.neuroscience.2012.05.057 22659565

[B27] Fischer A. Sananbenesi F. Wang X. Dobbin M. Tsai L. H. (2007). Recovery of learning and memory is associated with chromatin remodelling. Nature 447, 178–182 10.1038/nature05772 17468743

[B28] Gallistel C. R. Fairhurst S. Balsam P. (2004). The learning curve: implications of a quantitative analysis. Proc. Natl. Acad. Sci. U.S.A. 101, 13124–13131 10.1073/pnas.0404965101 15331782 PMC516535

[B29] Gao Z. Van Beugen B. J. De Zeeuw C. I. (2012). Distributed synergistic plasticity and cerebellar learning. Nat. Rev. Neurosci. 13, 619–635 10.1038/nrn3312 22895474

[B30] Ginsburg S. Jablonka E. (2009). Epigenetic learning in non-neural organisms. J. Biosci. 34, 633–646 10.1007/s12038-009-0081-8 19920348

[B31] Giuditta A. Kaplan B. B. Van Minnen J. Alvarez J. Koenig E. (2002). Axonal and presynaptic protein synthesis: new insights into the biology of the neuron. Trends Neurosci. 25, 400–404 10.1016/S0166-2236(02)02188-4 12127756

[B32] Glautier S. (2013). Revisiting the learning curve (once again). Front. Psychol. 4:982 10.3389/fpsyg.2013.00982 24421774 PMC3872722

[B33] Gräff J. Tsai L.-H. (2013). Histone acetylation: molecular mnemonics on the chromatin. Nat. Rev. Neurosci. 14, 97–111 10.1038/nrn3427 23324667

[B34] Graff J. Woldemichael B. T. Berchtold D. Dewarrat G. Mansuy I. M. (2012). Dynamic histone marks in the hippocampus and cortex facilitate memory consolidation. Nat. Commun. 3:991 10.1038/ncomms1997 22871810

[B35] Guan J. S. Haggarty S. J. Giacometti E. Dannenberg J. H. Joseph N. Gao J. (2009). HDAC2 negatively regulates memory formation and synaptic plasticity. Nature 459, 55–60 10.1038/nature07925 19424149 PMC3498958

[B36] Gupta S. Kim S. Y. Artis S. Molfese D. L. Schumacher A. Sweatt J. D. (2010). Histone methylation regulates memory formation. J. Neurosci. 30, 3589–3599 10.1523/JNEUROSCI.3732-09.2010 20219993 PMC2859898

[B37] Gupta-Agarwal S. Franklin A. V. Deramus T. Wheelock M. Davis R. L. Mcmahon L. L. (2012). G9a/GLP histone lysine dimethyltransferase complex activity in the hippocampus and the entorhinal cortex is required for gene activation and silencing during memory consolidation. J. Neurosci. 32, 5440–5453 10.1523/JNEUROSCI.0147-12.2012 22514307 PMC3332335

[B38] Han J. Li Y. Wang D. Wei C. Yang X. Sui N. (2010). Effect of 5-aza-2-deoxycytidine microinjecting into hippocampus and prelimbic cortex on acquisition and retrieval of cocaine-induced place preference in C57BL/6 mice. Eur. J. Pharmacol. 642, 93–98 10.1016/j.ejphar.2010.05.050 20550947

[B39] Hata K. Mizukami H. Sadakane O. Watakabe A. Ohtsuka M. Takaji M. (2013). DNA methylation and methyl-binding proteins control differential gene expression in distinct cortical areas of macaque monkey. J. Neurosci. 33, 19704–19714 10.1523/JNEUROSCI.2355-13.2013 24336734 PMC6618758

[B40] Hawk J. D. Florian C. Abel T. (2011). Post-training intrahippocampal inhibition of class I histone deacetylases enhances long-term object-location memory. Learn. Mem. 18, 367–370 10.1101/lm.2097411 21576516 PMC3101774

[B41] Holtmaat A. Svoboda K. (2009). Experience-dependent structural synaptic plasticity in the mammalian brain. Nat. Rev. Neurosci. 10, 647–658 10.1038/nrn2699 19693029

[B42] Ibrahim A. E. Arends M. J. Silva A. L. Wyllie A. H. Greger L. Ito Y. (2011). Sequential DNA methylation changes are associated with DNMT3B overexpression in colorectal neoplastic progression. Gut 60, 499–508 10.1136/gut.2010.223602 21068132

[B43] Jablonka E. Lamb M. (2005/2014). Evolution in Four Dimensions: Genetic, Epigenetic, Behavioral, and Symbolic Variation in the History of Life. Cambridge, MA: MIT press

[B44] Kemenes I. Straub V. A. Nikitin E. S. Staras K. O'Shea M. Kemenes G. (2006). Role of delayed nonsynaptic neuronal plasticity in long-term associative memory. Curr. Biol. 16, 1269–1279 10.1016/j.cub.2006.05.049 16824916

[B45] Konopka W. Kiryk A. Novak M. Herwerth M. Parkitna J. R. Wawrzyniak M. (2010). MicroRNA loss enhances learning and memory in mice. J. Neurosci. 30, 14835–14842 10.1523/JNEUROSCI.3030-10.2010 21048142 PMC6633640

[B46] Lachenal G. Pernet-Gallay K. Chivet M. Hemming F. J. Belly A. Bodon G. (2011). Release of exosomes from differentiated neurons and its regulation by synaptic glutamatergic activity. Mol. Cell. Neurosci. 46, 409–418 10.1016/j.mcn.2010.11.004 21111824

[B47] Ladd-Acosta C. Hansen K. D. Briem E. Fallin M. D. Kaufmann W. E. Feinberg A. P. (2013). Common DNA methylation alterations in multiple brain regions in autism. Mol. Psychiatry. [Epub ahead of print]. 10.1038/mp.2013.114 23999529 PMC4184909

[B48] Lai C. P. Breakefield X. O. (2012). Role of exosomes/microvesicles in the nervous system and use in emerging therapies. Front. Physiol. 3:228 10.3389/fphys.2012.00228 22754538 PMC3384085

[B49] Lasalle J. M. Powell W. T. Yasui D. H. (2013). Epigenetic layers and players underlying neurodevelopment. Trends Neurosci. 36, 460–470 10.1016/j.tins.2013.05.001 23731492 PMC3735843

[B50] Leslie J. H. Nedivi E. (2011). Activity-regulated genes as mediators of neural circuit plasticity. Prog. Neurobiol. 94, 223–237 10.1016/j.pneurobio.2011.05.002 21601615 PMC3134580

[B51] Levenson J. M. O'Riordan K. J. Brown K. D. Trinh M. A. Molfese D. L. Sweatt J. D. (2004). Regulation of histone acetylation during memory formation in the hippocampus. J. Biol. Chem. 279, 40545–40559 10.1074/jbc.M402229200 15273246

[B52] Levenson J. M. Roth T. L. Lubin F. D. Miller C. A. Huang I. C. Desai P. (2006). Evidence that DNA (cytosine-5) methyltransferase regulates synaptic plasticity in the hippocampus. J. Biol. Chem. 281, 15763–15773 10.1074/jbc.M511767200 16606618

[B53] Li H. Zhong X. Chau K. F. Williams E. C. Chang Q. (2011). Loss of activity-induced phosphorylation of MeCP2 enhances synaptogenesis, LTP and spatial memory. Nat. Neurosci. 14, 1001–1008 10.1038/nn.2866 21765426 PMC3273496

[B54] Li L. C. Okino S. T. Zhao H. Pookot D. Place R. F. Urakami S. (2006). Small dsRNAs induce transcriptional activation in human cells. Proc. Natl. Acad. Sci. U.S.A. 103, 17337–17342 10.1073/pnas.0607015103 17085592 PMC1859931

[B55] Li X. Wei W. Zhao Q. Y. Widagdo J. Baker-Andresen D. Flavell C. R. (2014). Neocortical Tet3-mediated accumulation of 5-hydroxymethylcytosine promotes rapid behavioral adaptation. Proc. Natl. Acad. Sci. U.S.A. 111, 7120–7125 10.1073/pnas.1318906111 24757058 PMC4024925

[B56] Lisman J. Schulman H. Cline H. (2002). The molecular basis of CaMKII function in synaptic and behavioural memory. Nat. Rev. Neurosci. 3, 175–190 10.1038/nrn753 11994750

[B57] Lister R. Mukamel E. A. Nery J. R. Urich M. Puddifoot C. A. Johnson N. D. (2013). Global epigenomic reconfiguration during mammalian brain development. Science 341:1237905 10.1126/science.1237905 23828890 PMC3785061

[B58] Lockett G. A. Helliwell P. Maleszka R. (2010). Involvement of DNA methylation in memory processing in the honey bee. Neuroreport 21, 812–816 10.1097/WNR.0b013e32833ce5be 20571459

[B59] Lu H. Liu X. Deng Y. Qing H. (2013). DNA methylation, a hand behind neurodegenerative diseases. Front. Aging Neurosci. 5:85 10.3389/fnagi.2013.00085 24367332 PMC3851782

[B60] Lubin F. D. Roth T. L. Sweatt J. D. (2008). Epigenetic regulation of BDNF gene transcription in the consolidation of fear memory. J. Neurosci. 28, 10576–10586 10.1523/JNEUROSCI.1786-08.2008 18923034 PMC3312036

[B61] Mahan A. L. Mou L. Shah N. Hu J. H. Worley P. F. Ressler K. J. (2012). Epigenetic modulation of Homer1a transcription regulation in amygdala and hippocampus with pavlovian fear conditioning. J. Neurosci. 32, 4651–4659 10.1523/JNEUROSCI.3308-11.2012 22457511 PMC3329762

[B62] Mastroeni D. Grover A. Delvaux E. Whiteside C. Coleman P. D. Rogers J. (2010). Epigenetic changes in Alzheimer's disease: decrements in DNA methylation. Neurobiol. Aging 31, 2025–2037 10.1016/j.neurobiolaging.2008.12.005 19117641 PMC2962691

[B63] Mehta D. Klengel T. Conneely K. N. Smith A. K. Altmann A. Pace T. W. (2013). Childhood maltreatment is associated with distinct genomic and epigenetic profiles in posttraumatic stress disorder. Proc. Natl. Acad. Sci. U.S.A. 110, 8302–8307 10.1073/pnas.1217750110 23630272 PMC3657772

[B64] Melnyk S. Fuchs G. J. Schulz E. Lopez M. Kahler S. G. Fussell J. J. (2012). Metabolic imbalance associated with methylation dysregulation and oxidative damage in children with autism. J. Autism Dev. Disord. 42, 367–377 10.1007/s10803-011-1260-7 21519954 PMC3342663

[B65] Miller C. A. Gavin C. F. White J. A. Parrish R. R. Honasoge A. Yancey C. R. (2010). Cortical DNA methylation maintains remote memory. Nat. Neurosci. 13, 664–666 10.1038/nn.2560 20495557 PMC3043549

[B66] Miller C. A. Sweatt J. D. (2007). Covalent modification of DNA regulates memory formation. Neuron 53, 857–869 10.1016/j.neuron.2007.02.022 17359920

[B67] Monsey M. S. Ota K. T. Akingbade I. F. Hong E. S. Schafe G. E. (2011). Epigenetic alterations are critical for fear memory consolidation and synaptic plasticity in the lateral amygdala. PLoS ONE 6:e19958 10.1371/journal.pone.0019958 21625500 PMC3098856

[B68] Rajasethupathy P. Antonov I. Sheridan R. Frey S. Sander C. Tuschl T. (2012). A Role for Neuronal piRNAs in the epigenetic control of memory-related synaptic plasticity. Cell 149, 693–707 10.1016/j.cell.2012.02.057 22541438 PMC3442366

[B68a] Rescorla R. A. Wagner A. R. (1972). A theory of Pavlovian conditioning: Variations in the effectiveness of reinforcement and nonreinforcement, in Classical Conditioning II: Current Research and Theory, eds Black A. H. Prokasy W. F. (New York, NY: Appleton-Century-Crofts), 64–99

[B69] Schacter D. L. (2001). Forgotten Ideas, Neglected Pioneers: Richard Semon and the Story of Memory. Philadelphia, PA: Psychology Press

[B69a] Semon R. (1921). The Mneme. London: Allen and Unwin 1st Edn. 1904; Translation of the 3rd Edn. 1911 by Louis Simon.

[B69b] Semon R (1923). Mnemic Psychology. London: Allen and Unwin Translation by Vernon Lee of the 1909 original edition.

[B70] Shorter J. Lindquist S. (2005). Prions as adaptive conduits of memory and inheritance. Nat. Rev. Genet. 6, 435–450 10.1038/nrg1616 15931169

[B71] Smith Z. D. Meissner A. (2013). DNA methylation: roles in mammalian development. Nat. Rev. Genet. 14, 204–220 10.1038/nrg3354 23400093

[B72] Solomon M. Smith A. C. Frank M. J. Ly S. Carter C. S. (2011). Probabilistic reinforcement learning in adults with autism spectrum disorders. Autism Res. 4, 109–120 10.1002/aur.177 21425243 PMC5538882

[B73] Spadaro P. A. Bredy T. W. (2012). Emerging role of non-coding RNA in neural plasticity, cognitive function, and neuropsychiatric disorders. Front. Genet. 3:132 10.3389/fgene.2012.00132 22811697 PMC3395882

[B74] Sui L. Wang Y. Ju L. H. Chen M. (2012). Epigenetic regulation of reelin and brain-derived neurotrophic factor genes in long-term potentiation in rat medial prefrontal cortex. Neurobiol. Learn. Mem. 97, 425–440 10.1016/j.nlm.2012.03.007 22469747

[B75] Talbert P. B. Henikoff S. (2010). Histone variants–ancient wrap artists of the epigenome. Nat. Rev. Mol. Cell Biol. 11, 264–275 10.1038/nrm2861 20197778

[B76] Thorndike E. (1898). Some experiments on animal intelligence. Science 7, 818–824 10.1126/science.7.181.818 17769765

[B77] Uddin M. Aiello A. E. Wildman D. E. Koenen K. C. Pawelec G. De Los Santos R. (2010). Epigenetic and immune function profiles associated with posttraumatic stress disorder. Proc. Natl. Acad. Sci. U.S.A. 107, 9470–9475 10.1073/pnas.0910794107 20439746 PMC2889041

[B78] Walker J. Fowler S. Miller D. Sun A. Weisman G. Wood W. (2011). Spatial learning and memory impairment and increased locomotion in a transgenic amyloid precursor protein mouse model of Alzheimer's disease. Behav. Brain Res. 222, 169–175 10.1016/j.bbr.2011.03.049 21443906

[B79] Wang W. H. Cheng L. C. Pan F. Y. Xue B. Wang D. Y. Chen Z. (2011). Intracellular trafficking of histone deacetylase 4 regulates long−term memory formation. Anat. Rec. 294, 1025–1034 10.1002/ar.21389 21542139

[B80] Ward B. McGuinness L. Akerman C. J. Fine A. Bliss T. V. Emptage N. J. (2006). State-dependent mechanisms of LTP expression revealed by optical quantal analysis. Neuron 52, 649–661 10.1016/j.neuron.2006.10.007 17114049

[B81] Wu H. Tao J. Chen P. J. Shahab A. Ge W. Hart R. P. (2010). Genome-wide analysis reveals methyl-CpG-binding protein 2-dependent regulation of microRNAs in a mouse model of Rett syndrome. Proc. Natl. Acad. Sci. U.S.A. 107, 18161–18166 10.1073/pnas.1005595107 20921386 PMC2964235

[B82] Yehuda R. Bierer L. M. (2009). The relevance of epigenetics to PTSD: implications for the DSM-V. J. Trauma Stress 22, 427–434 10.1002/jts.20448 19813242 PMC2891396

[B83] Yu F. Zingler N. Schumann G. Strätling W. H. (2001). Methyl-CpG-binding protein 2 represses LINE-1 expression and retrotransposition but not Alu transcription. Nucleic Acids Res. 29, 4493–4501 10.1093/nar/29.21.4493 11691937 PMC60185

[B84] Yu N. K. Baek S. H. Kaang B. K. (2011). DNA methylation-mediated control of learning and memory. Mol. Brain 4:5 10.1186/1756-6606-4-5 21247469 PMC3033800

[B85] Zlomuzica A. Dere D. Machulska A. Adolph D. Dere E. Margraf J. (2014). Episodic memories in anxiety disorders: clinical implications. Front. Behav. Neurosci. 8:131 10.3389/fnbeh.2014.00131 24795583 PMC4005957

[B86] Zovkic I. B. Guzman-Karlsson M. C. Sweatt J. D. (2013). Epigenetic regulation of memory formation and maintenance. Learn. Mem. 20, 61–74 10.1101/lm.026575.112 23322554 PMC3549063

[B87] Zwissler B. Hauswald A. Koessler S. Ertl V. Pfeiffer A. Wohrmann C. (2012). Memory control in post-traumatic stress disorder: evidence from item method directed forgetting in civil war victims in Northern Uganda. Psychol. Med. 42, 1283–1291 10.1017/S0033291711002273 22011378

